# Doxycycline decelerates aging in progeria mice

**DOI:** 10.1111/acel.14188

**Published:** 2024-04-30

**Authors:** Ming Wang, Jie Zhang, Jiangping Qiu, Xuan Ma, Chenzhong Xu, Qiuhuan Wu, Shaojun Xing, Xinchun Chen, Baohua Liu

**Affiliations:** ^1^ Shenzhen Key Laboratory for Systemic Aging and Intervention (SKL‐SAI), Marshall Laboratory of Biomedical Engineering, International Cancer Center, School of Basic Medical Sciences Shenzhen University Medical School Shenzhen China; ^2^ Guangdong Provincial Key Laboratory of Regional Immunity and Diseases, School of Basic Medical Sciences Shenzhen University Medical School Shenzhen China

**Keywords:** aging, doxycycline, Hutchinson‐Gilford progeria syndrome (HGPS), IL6, tubulin acetylation

## Abstract

Beyond the antimicrobial activity, doxycycline (DOX) exhibits longevity‐promoting effect in nematodes, while its effect on mammals is unclear. Here, we applied a mouse model of Hutchinson‐Gilford progeria syndrome (HGPS), *Zmpste24* knockout (KO) mice, and analyzed the antiaging effect of DOX. We found that the DOX treatment prolongs lifespan and ameliorates progeroid features of *Zmpste24* KO mice, including the decline of body and tissue weight, exercise capacity and cortical bone density, and the shortened colon length. DOX treatment alleviates the abnormal nuclear envelope in multiple tissues, and attenuates cellular senescence and cell death of *Zmpste24* KO and HGPS fibroblasts. DOX downregulates the level of proinflammatory IL6 in both serum and tissues. Moreover, the elevated α‐tubulin (K40) acetylation mediated by NAT10 in progeria, is rescued by DOX treatment in the aorta tissues in *Zmpste24* KO mice and fibroblasts. Collectively, our study uncovers that DOX can decelerate aging in progeria mice via counteracting IL6 expression and NAT10‐mediated acetylation of α‐tubulin.

## Graphical abstract

Doxycycline decelerates aging in progeria mice and alleviates cell senescence in Zmpste24 KO MEFs and HGPS fibroblasts, probably via counteracting IL6 expression and NAT10‐mediated tubulin acetylation. This study uncovers the anti‐premature aging effect (“new function”) of the doxycycline (“old” drug), highlighting a safe and affordable therapeutic drug for HGPS.

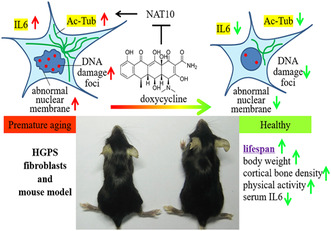

Hutchinson‐Gilford progeria syndrome (HGPS) is a rare human disease caused by a truncated mutant of lamin A precursor, called progerin (Hamczyk et al., 2018). Loss of *Zmpste24*, which encodes *Zmpste24*, leads to accumulation of the unprocessed precursor of lamin A, prelamin A, and premature aging phenotypes in mice, recapitulating HGPS. Thus, *Zmpste24* knockout (KO) mice have been widely used to investigate the pathogenesis of HGPS and for preclinical testing and therapies (Lai & Wong, 2020). Though achievements have been made in the past two decades, more efforts are needed to seek satisfactory approaches for HGPS therapy.

Doxycycline (DOX) elicits activity against a broad range of bacteria via inhibiting bacterial ribosomes and has been clinically used for over 50 years (Singh et al., 2021). DOX also exerts non‐classic function such as anti‐inflammatory, antineoplastic, and neuroprotective effects (Singh et al., 2021). A report disclosed that DOX can prolong the lifespan of *C. elegans* via stimulating the mitochondrial unfold protein response (UPR^mt^), a conserved pathway from worms to mammals (Houtkooper et al., 2013). This arouses our great interest to investigate the DOX effect on the mammalian lifespan. To that end, *Zmpste24* KO mice were orally administered with 1 mg/mL of DOX in drinking water starting from 1 month of age. Notably, DOX administration alleviated the decline of body weight (Figure 1a,b), and tissue weight (Figure 1c and Figure S1a) in both male and female progeria mice. Moreover, DOX treatment prolonged the medium and maximum lifespan of *Zmpste24* KO mice (Figure 1d). Though the DOX treatment elicited ignorable effect on the rib fractures (Figure S1b), it improved the thigh cortical bone density (Figure S1c). As determined by treadmill test, DOX feeding significantly enhanced the physical activity of progeria mice (Figure 1e). DOX also significantly prevented the shortening of colon length (Figure S1d), while having negligible effect on the structure of gastrointestinal tract (Figure S1e,f).

**FIGURE 1:**
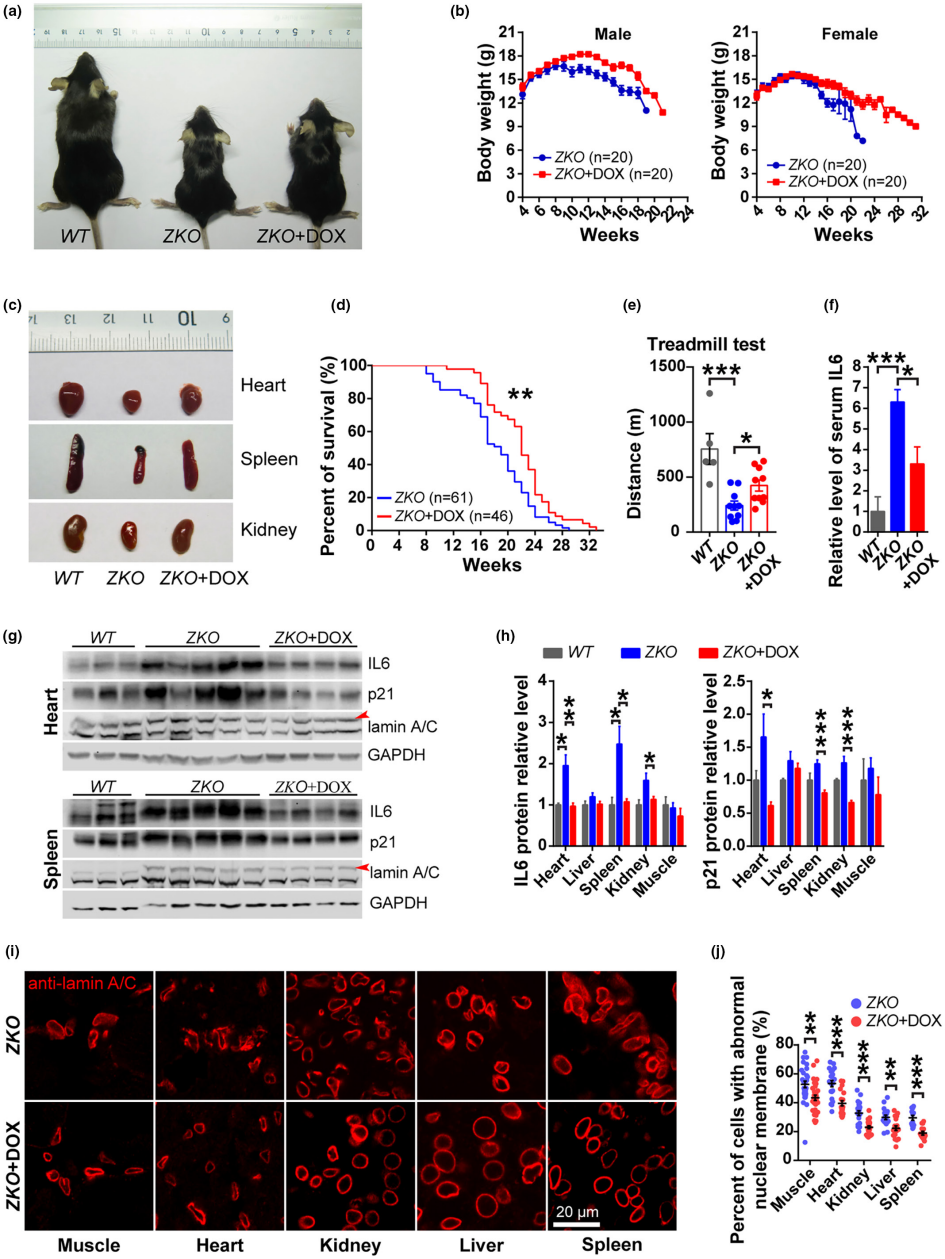
Doxycycline alleviates the aging features of *Zmpste24*‐deficient progeria mice. (a) The appearance of wild‐type (WT), *Zmpste24* KO (ZKO) and doxycycline (DOX) treated *Zmpste24* KO (ZKO + DOX) mice at 4 months of age. (b) The body weight of male and female ZKO mice treated with or without DOX. (c) The appearance of tissues from WT, ZKO and ZKO + DOX mice at 4 months of age. (d) The lifespan of ZKO mice treated with or without DOX. (e) The treadmill test of WT (*n* = 5), ZKO (*n* = 10) and ZKO + DOX (*n* = 10) mice at 4 months of age. (f) The ELISA analysis of serum IL6 level of WT (*n* = 8), ZKO (*n* = 8) and ZKO + DOX (*n* = 10) mice at 4 months of age. (g) The western blotting analysis of IL6, p21 and lamin A/C protein expression in heart and spleen tissues from WT, ZKO and ZKO + DOX mice at 4 months of age. (h) The quantitative analysis of IL6 and p21 protein expression in tissues. (i) The representative figures of immunofluorescence (IF) staining with anti‐lamin A/C antibody in tissues of ZKO and ZKO + DOX mice at 4 months of age. Bar, 20 μm. (j) The statistical analysis of the IF staining results as shown in (i). * *p* < 0.05, ** *p* < 0.01, *** *p* < 0.001. *n*, mice number.

The chronic inflammation is considered as a critical driver of aging (Lopez‐Otin et al., 2023). Pro‐inflammation cytokine Interleukin 6 (IL6) is elevated in HGPS cells, *Zmpste24* KO mice as well as *Lmna* ^G609G/G609G^ mice (Osorio et al., 2012; Squarzoni et al., 2021). Treatment with tocilizumab, an anti‐IL6R mono‐antibody, prolongs lifespan, and ameliorates aging symptoms of *Lmna* ^G609G/G609G^ mice via blocking the IL6 signaling (Squarzoni et al., 2021). Increasing evidence has suggested that DOX exerts anti‐inflammation effect by suppressing IL6 production (Di Caprio et al., 2015; Henehan et al., 2017). We therefore examined whether the longevity‐promoting effect of DOX in *Zmpste24* KO mice is mediated by suppressing IL6 production. To that end, we examined the serum level of IL6 by ELISA. Compared to wild type (WT) mice, the serum level of IL6 in progeria mice was 6.3 times increased, which was then notably decreased upon DOX treatment (Figure 1f). Further, we investigated the IL6 expression in individual tissues by western blotting. As shown, IL6 protein level was significantly increased in the heart, spleen and kidney tissues of progeria mice, which was recovered to a comparable level to that in WT after the DOX feeding (Figure 1g,h and Figure S1g). Meanwhile, the expression level of cell senescence biomarker p21^Wif1^ was increased in all tested tissues in *Zmpste24* KO mice compared to WT mice, which was restored after the DOX treatment (Figure 1g,h and Figure S1g). In addition, DOX treatment largely alleviated the nuclear membrane morphology and the percentage of cells with abnormal nuclear membrane was notably decreased in these tissues (Figure 1i,j).

We next examined the DOX‐rescue effect on cellular senescence. To that end, in vitro cultured mouse embryonic fibroblasts (MEFs) isolated from WT and *Zmpste24* KO mice were treated with 1 μg/mL of DOX. As shown, DOX had ignorable effect on the mRNA levels of the senescence biomarkers *p16* ^*Ink4a*^ and *p21* ^*Wif1*^ in both WT and *Zmpste24* KO MEFs. In contrast, the mRNA levels of *Il6* and *Il1b* in *Zmpste24* KO MEFs were significantly reduced upon DOX treatment, but not in WT MEFs (Figure 2a and Figure S2a). Western blotting results showed that the protein levels of IL6 and p16^Ink4a^ were 23.96% and 16.95% decreased respectively in *Zmpste24* KO cells treated with DOX (Figure S2b). Meanwhile, DOX treatment significantly reduced the percent of abnormal nuclear membrane in *Zmpste24* KO MEFs, which had ignorable effect in WT MEFs (Figure 2b and Figure S2c). Genomic instability is a hallmark of HGPS and *Zmpste24* KO cells (Liu et al., 2005; Wang et al., 2020). Increased DNA damage foci (53BP1 staining) were observed in *Zmpste24* KO cells compared to WT, and DOX treatment reduced the foci number per cell in KO MEFs, but had ignorable effect in WT (Figure 2c and Figure S2d). Meanwhile, percent cells with positive senescence‐associated β‐galactosidase activity (SA‐β‐Gal) staining were significantly decreased upon DOX treatment (Figure 2d). Of note, DOX treatment had negligible effect on WT MEFs (Figure S2e). Together, these data indicate that DOX alleviates cellular senescence of *Zmpste24* KO MEFs by an intrinsic mechanism.

**FIGURE 2:**
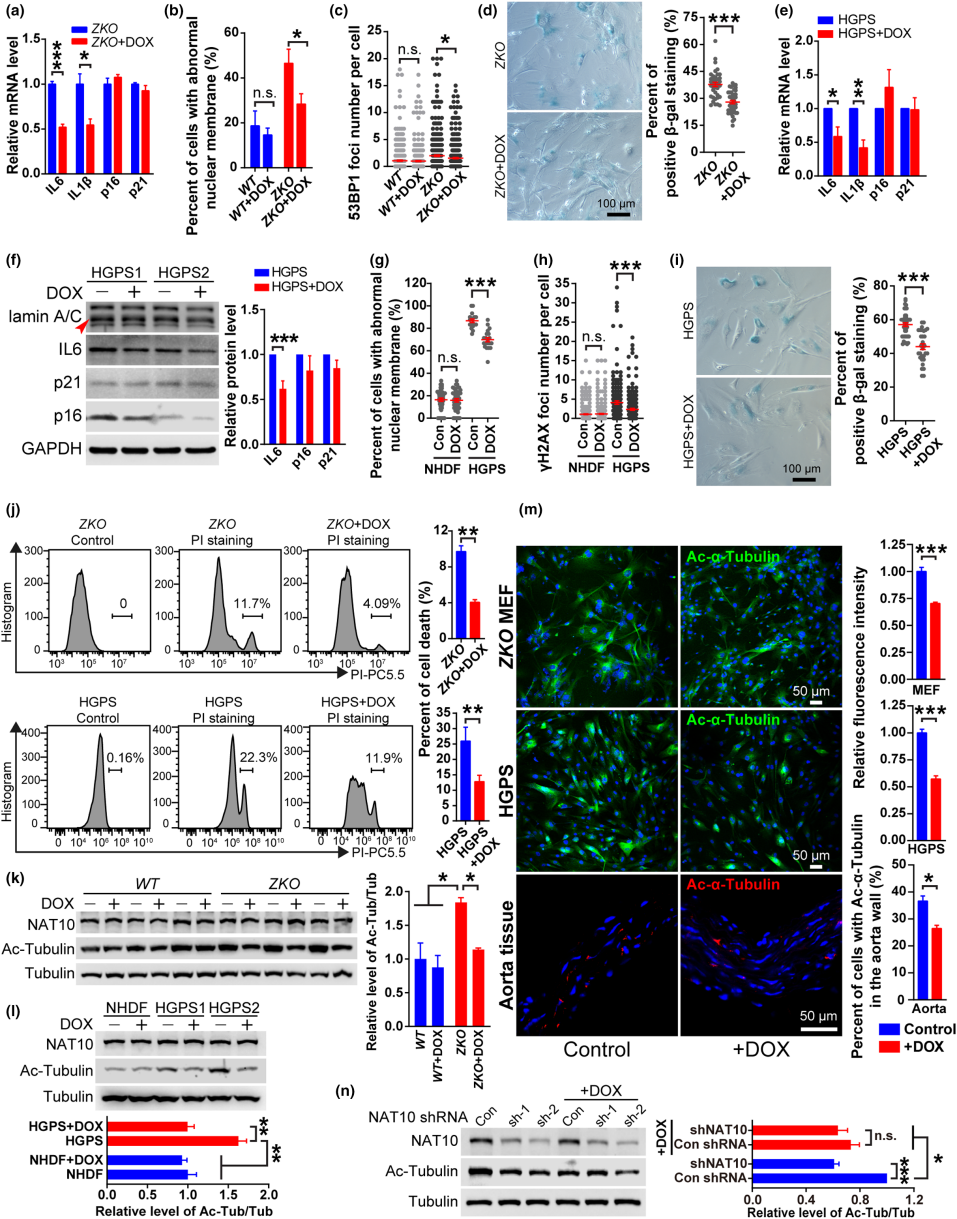
Doxycycline alleviates the cell senescence of *Zmpste24* KO MEF and HGPS fibroblasts in vitro. (a) The q‐RT‐PCR analysis of mRNA expression of *Il6*, *Il1b*, *p16* and *p21* in ZKO MEF cells with or without DOX treatment. (b,c) The statistical analysis of abnormal nuclear membrane (b) and the 53BP1 foci number (c) in WT or ZKO MEF cells with or without DOX treatment. (d) The SA‐β‐Gal staining analysis of ZKO MEF cells at passage 8 with or without DOX treatment. Bar, 100 μm. (e) The q‐RT‐PCR analysis of mRNA expression of *Il6*, *ILlb*, *p16* and *p21* in HGPS skin fibroblasts with or without DOX treatment. (f) The western blotting analysis of lamin A/C, IL6, p16 and p21 protein expression in HGPS cells with or without DOX treatment. HGPS1, HGADFN122; HGPS2, HGADFN169. (g,h) The statistical analysis of abnormal nuclear membrane (g) and the γH2AX foci number (h) in normal human dermal fibroblasts (NHDFs) and HGPS skin fibroblasts with or without DOX treatment. (i) The SA‐β‐Gal staining analysis of HGPS fibroblasts at passage 29 with or without DOX treatment. Bar, 100 μm. (j) The FACS analysis of cell death (PI staining) in ZKO MEF cells and HGPS fibroblasts with or without DOX treatment. (k,l) The western blotting analysis of NAT10 and Ac‐Tubulin protein expression in WT and ZKO MEF cells (k), NHDFs and HGPS fibroblasts (l) with or without DOX treatment. (m) The representative figures of immunofluorescence (IF) staining with anti‐Ac‐α‐Tubulin antibody in ZKO MEF cells, HGPS fibroblasts and aorta tissue from ZKO mice treated with or without DOX. Bar, 50 μm. (n) The analysis of Ac‐Tubulin protein expression in HGPS cells transfected with control (Con) or NAT10 shRNAs, and treated with or without DOX. n.s., nonsignificant. * *p* < 0.05, ** *p* < 0.01, *** *p* < 0.001.

The skin fibroblasts from HGPS patients (referred to as HGPS cells) and healthy individuals (NHDFs) were cultured in vitro and treated with DOX at late passages. As shown, DOX treatment had ignorable effect on mRNA levels of *p16*, *p21*, *Il6* and *Il1b* in NHDFs (Figure S2f). In contrast, upon the DOX treatment, the *Il6* and *Il1b* mRNA levels in HGPS cells were significantly reduced (Figure 2e). Meanwhile, upon DOX treatment, the protein levels of IL6, p16, and p21 were about 38.15%, 17.85% and 15.27% decreased respectively in HGPS cells (Figure 2f), while the changes were almost ignorable in NHDFs (Figure S2g). DOX treatment also significantly improved the abnormal nuclear membrane and alleviated genomic instability (indicated by γH2AX foci) in HGPS cells but not in NHDFs (Figure 2g,h and Figure S2h,i). Meanwhile, upon the DOX treatment, the percentage of positive cells with SA‐β‐Gal staining was significantly decreased in HGPS cells, but not in NHDFs (Figure 2i and Figure S2j). As determined by PI staining and flow cytometry, around 9.7% *Zmpste24* KO MEFs and 25.9% HGPS cells underwent cell death, which were reduced to 4.05% and 12.79% respectively after DOX treatment (Figure 2j and Figure S2k). However, DOX elicited ignorable effect on the cell death of WT MEFs and NHDFs (Figure S2l). These data affirm that DOX alleviates premature senescence.

UPR^mt^ mediates lifespan‐extension of *C. elegans* treated with DOX (Houtkooper et al., 2013). This prompted us to examine whether the same mechanism underlines the DOX‐rescue effect in progeria mice. In the liver tissues of WT mice, the protein level of mitochondria protein mtCO1 declined significantly upon DOX treatment, affirming that DOX effectively inhibited the mitochondrial protein synthesis (Figure S2m,n). In contrast, DOX exhibited ignorable effect on the expression of HSP60 and LONP1, two key factors in UPR^mt^ pathway, in the liver tissues of both WT and *Zmpste24* KO mice (Figure S2m,n). These results suggest that DOX exerts antiaging effect via UPR^mt^‐independent pathway in *Zmpste24* KO mice. Notably, the protein level of mtCO1 in *Zmpste24* KO livers was lower than that in WT (Figure S2m,n).

*N*‐acetyltransferase 10 (NAT10), a lysine acetyltransferase, acetylates α‐tubulin, p53, histone and RNA (Larrieu et al., 2014; Larrieu et al., 2018). NAT10‐mediated α‐tubulin (K40) acetylation (Ac‐α‐tubulin) enhances microtubule stabilization, thereby disturbing the nucleocytoplasmic shutting of cargos and causing cell dysfunction, and inhibition of NAT10 ameliorates aging features and promotes healthspan in progeria mouse model (Balmus et al., 2018; Larrieu et al., 2018). Compared with WT MEFs and NHDFs, the acetylation level of α‐tubulin (K40) were significantly increased in *Zmpste24* KO and HGPS fibroblasts respectively, while the NAT10 level were comparable (Figure 2k,l and Figure S2o,p). Interestingly, DOX treatment significantly reduced the acetylation of α‐tubulin in *Zmpste24* KO and HGPS fibroblasts, and aorta tissues of *Zmpste24* KO mice, while NAT10 protein level was unchanged (Figure 2k–m and Figure S2o,p). However, DOX elicited ignorable effect on the acetylation level of α‐tubulin in WT MEFs and NHDFs (Figure 2k,l). To test whether NAT10 mediates DOX‐inhibited α‐tubulin acetylation, HGPS cells were treated with *NAT10* shRNA and DOX. The results demonstrated that DOX and *NAT10* shRNA could independently reduce the acetylation level of α‐tubulin in HGPS cells, while the combined treatment did not further lower down the level (Figure 2n), suggesting an overlapped effect of DOX and *NAT10* shRNA on α‐tubulin acetylation. Thus, DOX prevents α‐tubulin acetylation partially through NAT10‐mediated pathway.

Collectively, we showed that the DOX treatment decelerates aging and extends lifespan in progeria mice. At the cellular level, DOX treatment alleviates senescence in HGPS and *Zmpste24* KO fibroblasts, while such effect is not obvious in normal human fibroblasts undergoing replicative senescence. At the molecular level, NAT10 acts as a potential molecular target of DOX and inhibition of NAT10 likely accounts for the antiaging effect of DOX in progeria mice. Notably, the sensitivity of UPR^mt^ to DOX treatment are different between progeria and WT mice, which suggests different mechanisms underlying the DOX effect in progeria and normal aging. Whether DOX can prevent natural aging merits further investigation. Mechanistically, in addition to NAT10 and IL6 pathway, other potential DOX‐targeted pathways can't be excluded. For instance, DOX has inhibitory activity on matrix metalloproteinase (MMPs), which may help alleviate the vascular smooth muscle cells loss caused by MMP13 dysfunction in progeria model (Pitrez et al., 2020). As an antibiotic, DOX might affect intestinal microbiota, the dysbiosis of which has been found in progeria patients and mice model (Barcena et al., 2019).

Of note, DOX reversed the level of p21 in *Zmpste24* KO tissues but not in HGPS cells (Figures 1g,h and 2f). One explanation is that certain type of cells with high level of p21 (p21^high^) is more sensitive to DOX in vivo than the in vitro cultured fibroblasts. Indeed, diversified types of p21^high^ cells are identified in mice tissues, for example, endothelial cells in heart (Wang et al., 2021). An alternate explanation is the downregulation of p21 in vivo is a systemic and secondary effect of DOX treatment. Though the DOX treatment improves several features of *Zmpste24* KO mice, the increase in lifespan is moderate, suggesting that the pathogenetic pathways underlying progeria were not all rescued. For instance, DOX treatment failed to rescue the bone abnormalities such as the cortical bone density and rib fracture (Figure S1b,c), which is highly relevant to mortality (Hoepelman et al., 2023). Nevertheless, our data uncovers the widely used antibiotic DOX as a safe and affordable therapeutic drug for HGPS.

## AUTHOR CONTRIBUTIONS

M.W., J. Z. and B.L. conceived and designed the experiments; M.W., J.Q., X.M. and Q.W. performed most of the experiments; J. Z. and C.X. provided technical support; S.X. and X.C. discussed the results; M.W. and B.L. analyzed the data and wrote the manuscript.

## CONFLICT OF INTEREST STATEMENT

The authors declare no conflict of interests.

## Supporting information


Appendix S1.

## Data Availability

Data sharing is not applicable to this article as no new data were created or analyzed in this study.
