# Supplementary material for: Doxycycline decelerates aging in progeria mice
Source: Aging Cell. 2024 Apr 30;23(7):e14188. doi: 10.1111/acel.14188 (PMC11258430; doi:10.1111/acel.14188)
Supplement: Supplementary file 1 — Appendix S1. [file ACEL-23-e14188-s001.docx]

**Supplementary information**

This file contains Experimental procedures, Supplementary Figure S1 and S2 with figure legends, Supplementary Tables 1 to 3.

**Doxycycline decelerates aging in progeria mice**

Ming Wang^1*^, Jie Zhang^1^, Jiangping Qiu^1^, Xuan Ma^1^, Chenzhong Xu^1^, Qiuhuan Wu^1^, Shaojun Xing^2^, Xinchun Chen^2^, Baohua Liu^1*^

^1^Shenzhen Key Laboratory for Systemic Aging and Intervention (SKL-SAI); Marshall Laboratory of Biomedical Engineering; International Cancer Center, School of Basic Medical Sciences, Shenzhen University Medical School, Shenzhen 518055, China

^2^Guangdong Provincial Key Laboratory of Regional Immunity and Diseases, School of Basic Medical Sciences, Shenzhen University Medical School, Shenzhen 518055, China

*Corresponding author. Email: ppliew@szu.edu.cn (B.L.), kuailewm@126.com (M.W.)

**EXPERIMENTAL PROCEDURES**

**Animal**

*Zmpste24* heterozygous mice were obtained from professor Zhongjun Zhou (Hong Kong University). *Zmpste24* heterozygous mice were intercrossed to produce *Zmpste24* KO mice. All mice were housed under specific pathogen free (SPF) conditions with a 12-hour light/dark cycle and fed with a standard chow diet. At 1 month after birth, body weight was recorded weekly. Doxycycline (DOX) was administrated via drinking water at the concentration of 1 mg/mL, and replaced weekly. Animal studies were handled in accordance with protocols approved by the Committee on the Use of Live Animals in Teaching and Research of Shenzhen University, China.

**Cell culture**

*Zmpste24* WT and KO mouse embryonic fibroblasts (MEF) were isolated form embryos at day 13.5 of gestation. The normal human dermal fibroblasts (NHDF) were isolated from the skin tissue of a healthy female donor with written informed consent as described in our previous study (Wang et al., 2020). HGPS fibroblasts, i.e., HGADFN122 and HGADFN169, referred to HGPS1 and HGPS2 respectively, were obtained from the Progeria Research Foundation (PRF). Cells were maintained in DMEM medium with FBS (10% FBS for MEF cells, 15% for NHDF and HGPS cells), and cultured at 37 °C in a humidified incubator with 5% CO_2_. For DOX treatment, cells were cultured in freshly prepared medium containing with DOX (final concentration to 1 μg/mL) and the medium was changed every day.

**Western blotting**

Tissues were disrupted using the tissue lyser and lysed in the RIPA buffer (20 mM Tris-HCl pH 7.5, 150 mM NaCl, 1 mM EDTA, 1 mM EGTA, 1% Na-deoxycholate, 0.1% SDS, 1% NP40, 1% Triton X-100, 1 mM PMSF, and protease inhibitor cocktail). Protein was quantified with the Pierce^TM^ BCA kit (23227, ThermoFisher) and equal quantity of protein samples were subjected to SDS-PAGE electrophoresis. After transferring the protein onto the PVDF membrane, 5% fat-free milk was used for membrane blocking. The membrane was then incubated with relative primary antibodies overnight at 4 °C. Thereafter, the HRP conjugated second antibodies were added and incubated at room temperature for 1 h before detection using the ECL substrate solution (34578, ThermoFisher). The primary antibodies used for western blotting are listed in the Supplementary Table 1.

**RNA extraction and quantitative RT-PCR**

Tissues were disrupted and lysed in TRIzol reagent (9109, Takara) using the tissue lyser. Total RNA was isolated and cDNA was synthesized from 2 μg RNA of each sample using the PrimeScript RT Master kit (RR036A, Takara). Quantitative PCR was performed on the qTOWER^3^ Analytik Jena (Germany) using the Hieff q-PCR SYBR Green Master Mix (11201, Yeasen). Relative gene expression was quantified by normalizing to *GAPDH*, *Actin*, or 18s rRNA values. Primers for RT-PCR are listed in the Supplementary Table 2.

**shRNA lenti-virus infection**

The scramble and human NAT10 shRNA were constructed into pLKO.1 lenti-virus backbone. Virus was packaged in HEK293T cells by transfecting shRNA together with packaging plasmids psPAX2 and pMD2.G. At 48h after transfection, the virus was harvested, filtered and diluted at 1:1 with fresh medium containing 8 mg/L of polybrene for cell infection.

**Treadmill test**

Treadmill exhaustion tests were performed using the motorized treadmill at an incline of 5° with 0.5 mA electrical stimulation (SA101B, SANS Bio Instrument). Mice were trained for 3 days as the 1^st^ day at the speed of 5 m/min for 2 min, the 2^nd^ day at the speed of 7 m/min for 2 min, and the 3^rd^ day at the speed of 9 m/min for 1 min. After 1 day of rest, mice were tested at an initial speed of 5 m/min, and then increased by 3 m/min every 3 min up to the maximum speed of 26 m/min. The traveled distance for each mouse was recorded.

**Senescence associated β-galactosidase (SA-β-Gal) staining**

SA-β-Gal staining was performed following the manufacturer’s instructions provided by the kit (C0602, Beyotime). Cells cultured in a 12-well plate were fixed and incubated with freshly prepared staining solution at 37 °C in a dry incubator for 15 h before analysis.

**Immunofluorescence staining**

Cells plated on the glass coverslip or tissue sections were fixed with 4% paraformaldehyde (PFA) for 5 min. After permeabilization with 0.25% Trion X-100 for 5 min and subsequent blocking with 3% BSA for 1 h, cells were incubated with primary antibodies (Supplementary Table 1) overnight at 4 °C. Then fluorescence conjugated secondary antibodies were added and incubated for another 1 h at room temperature. Photos were collected using the the DragonFly confocal imaging system (Andor) and fluorescent microscopy Axio Vert.A1 (Carl Zeiss).

**Hematoxylin-eosin (H&E) staining**

Paraffin-embedded sections of PFA-fixed colon tissues were dewaxed and rehydrated. Then the tissues were performed H&E staining in the order of hematoxylin staining, differentiation, bluing, dehydration, eosin staining, clearing and addition of cover-slip.

**ELISA analysis of serum IL6**

The Mouse serum IL6 levels were measured using the Sandwich ELISA Kit (CME0006, 4A Biotech) and assay was performed according to the manufacturer’s protocols. Signals were measured at the OD of 450 nm on the Microplate Reader (PerkinElmer, EnSpire), and standard curve was established to quantify the concentration of serum IL6.

**Bone density analysis**

The thigh bone of *Zmpste24* KO mice treated with or without DOX were harvested and fixed in 4% PFA at 4 °C overnight. The cortical bone density data were collected by micro-CT (Scanco Medical, μCT100).

**Flow cytometry analysis**

*Zmpste24* KO MEF cells and HGPS fibroblasts treated with or without DOX were harvested for PI staining (BL110A, Biosharp) according to the manufacturer’s protocols. Then the PE channel was used for flow cytometry analysis by CytoFLEX (Beckman). The data were analyzed using the FlowJo software (FlowJo LLC)

**Statistical analysis**

Statistical analyses were performed with Excel and GraphPad Prism 7 software. The log-rank (Mantel-Cox) test was used to analyze the survival data while other data were analyzed using unpaired or paired Student’s *t*-tests (two-sided). All experiments were performed with at least three biological replicates. The data are presented as the means ± SEM, and *P* < 0.05 was considered statistically significant.

**SUPPLEMENTARY FIGURES**

**Supplementary Figure S1**


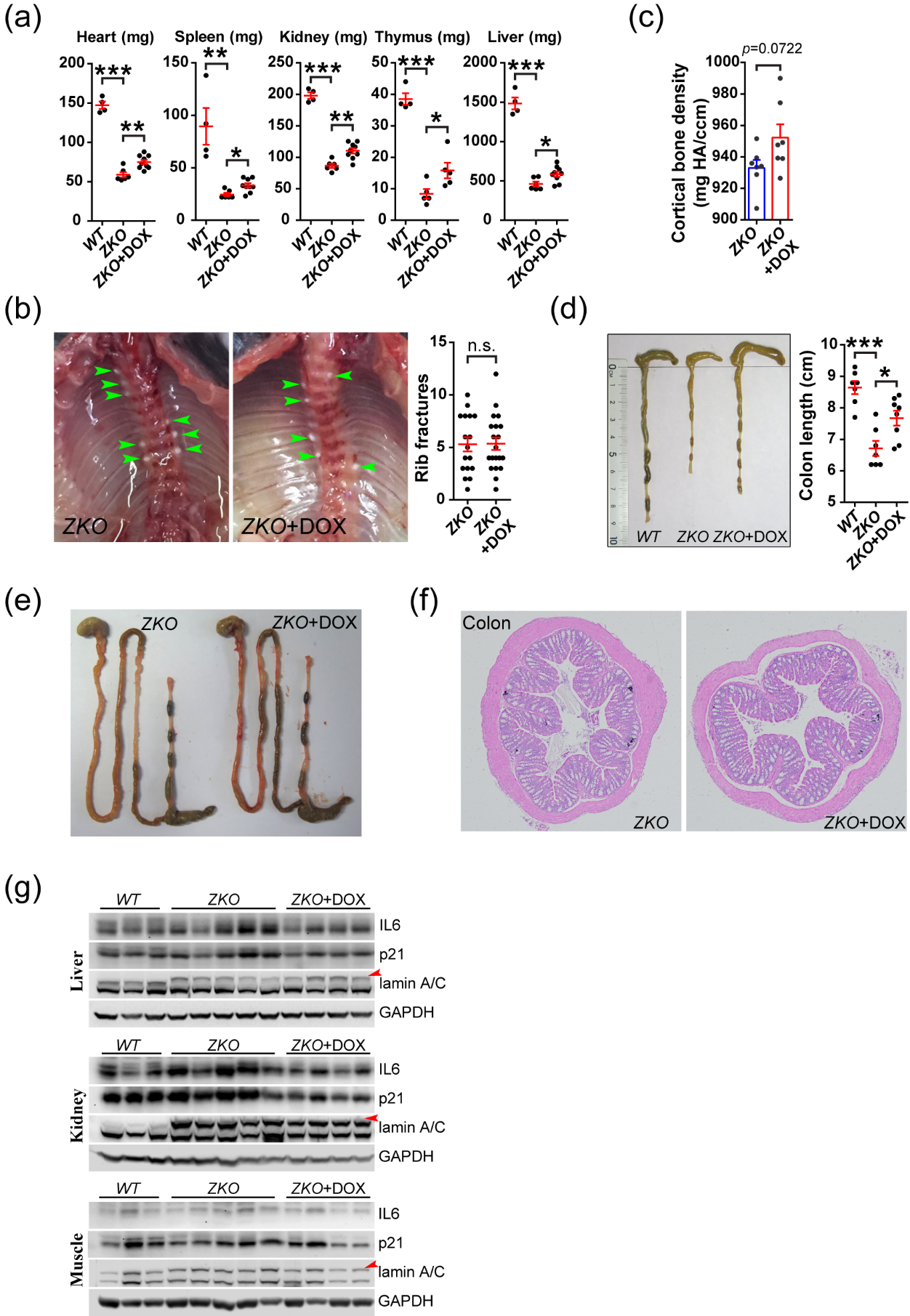


**Supplementary Figure S1** Doxycycline decelerates aging in *Zmpste24* KO mice. (a) The weight of tissues from wild-type (WT), *Zmpste24* KO (ZKO) and doxycycline (DOX) treated *Zmpste24* KO (ZKO+DOX) mice at 4 months of age. (b) The examination of rib fractures in ZKO mice at 4 months of age with or without DOX treatment. (c) The micro-CT analysis of the cortical bone density of ZKO and ZKO+DOX mice at 4 months of age. (d) The measurement of colon length of WT, ZKO and ZKO+DOX mice. (e) The appearance of gastrointestinal tract from ZKO mice at 4 months of age with or without DOX treatment. (f) The hematoxylin and eosin (HE) staining of colon from ZKO and ZKO+DOX mice at 4 months of age. (g) The western blotting analysis of IL6, p21 and lamin A/C protein expression in liver, kidney and muscle tissues of WT, ZKO and ZKO+DOX mice at 4 months of age. n.s., non-significant. * *p* < 0.05, ** *p* < 0.01, *** *p* < 0.001.

**Supplementary Figure S2**


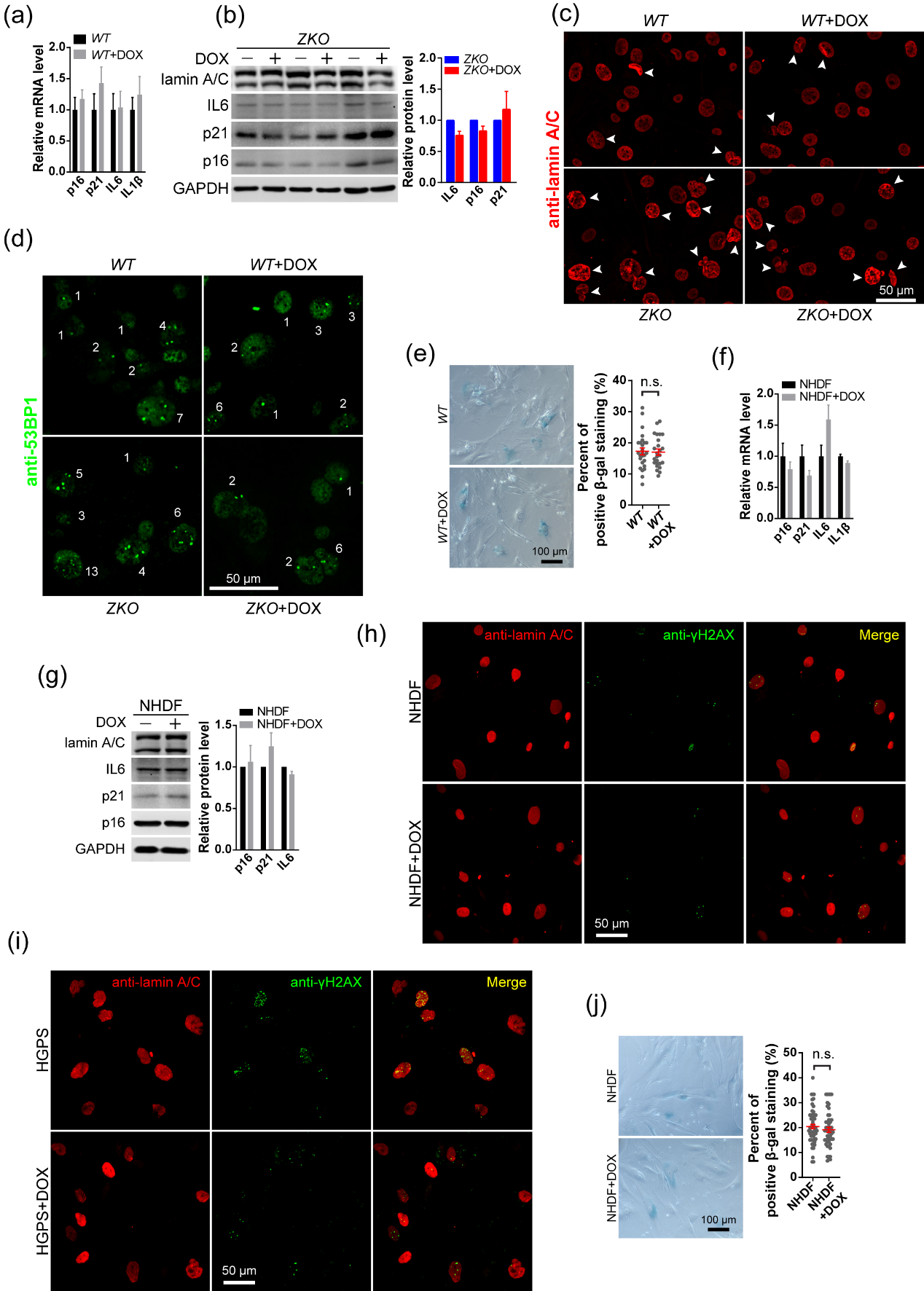


**Supplementary Figure S2**


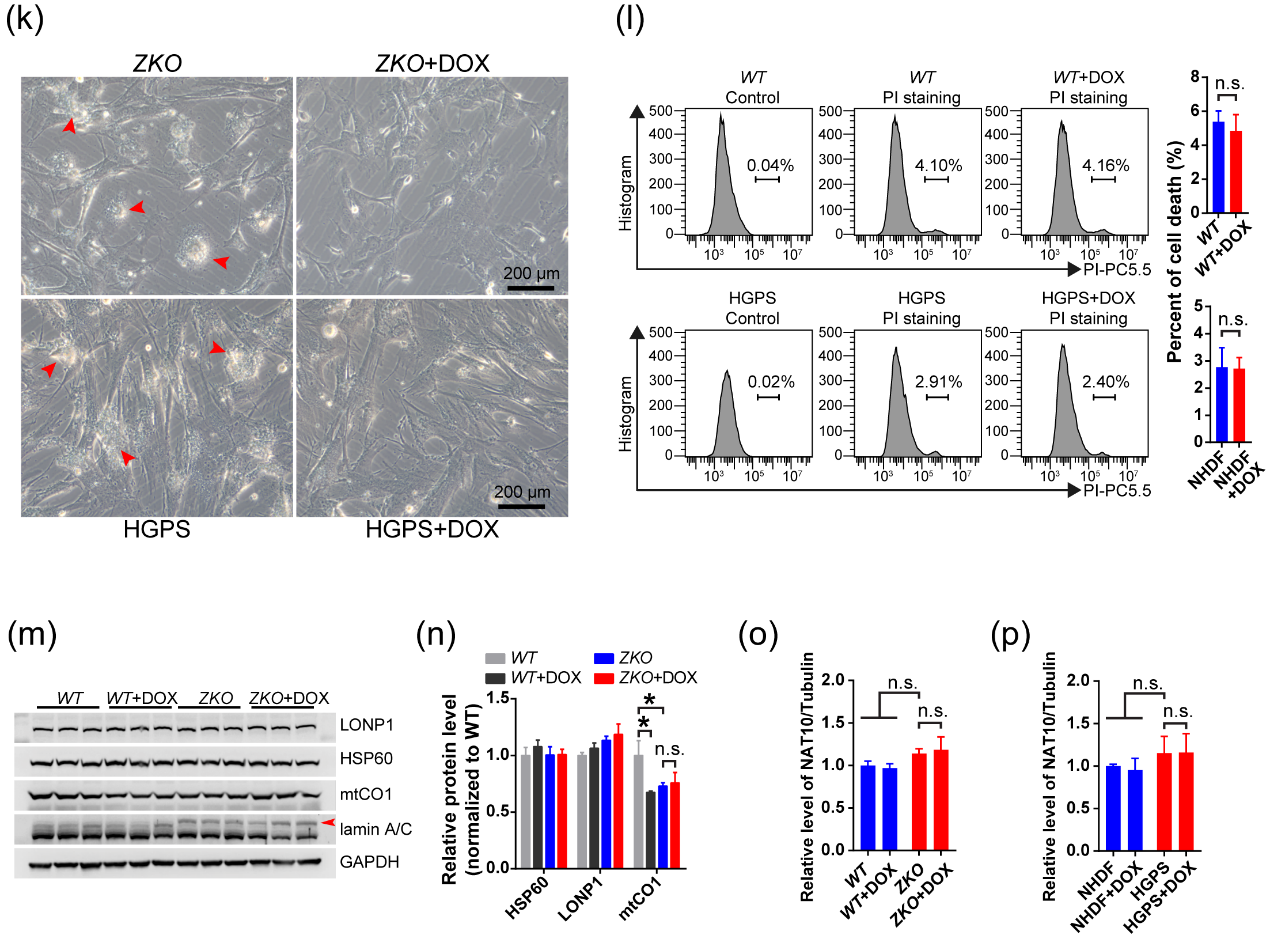


**Supplementary Figure S2** Doxycycline alleviates cellular senescence of *Zmpste24* KO MEF and HGPS fibroblasts. (a) The q-RT-PCR analysis of mRNA expression of *Il6*, *Il1b*, *p16* and *p21* in WT MEF cells with or without DOX treatment. (b) The western blotting analysis of lamin A/C, IL6, p16 and p21 protein expression in *Zmpste24* KO MEF cells with or without DOX treatment. (c,d) The representative figures of immunofluorescence (IF) staining with anti-lamin A/C (c) or anti-53BP1 (d) antibody in WT or *Zmpste24* KO MEF cells with or without DOX treatment. The white arrows indicate the abnormal nuclear membrane. Bar, 50 μm. (e) The SA-β-Gal staining analysis of WT MEF cells at passage 8 with or without DOX treatment. Bar, 100 μm. (f) The q-RT-PCR analysis of mRNA expression of *Il6*, *Il1b*, *p16* and *p21* in normal human dermal fibroblasts (NHDFs) with or without DOX treatment. (g) The western blotting analysis of lamin A/C, IL6, p16 and p21 protein expression in in NHDFs with or without DOX treatment. (h,i) The representative figures of immunofluorescence (IF) staining with anti-lamin A/C (red) and anti-γH2AX (green) antibodies in HGPS skin fibroblasts (h) and NHDFs (i) with or without DOX treatment. Bar, 50 μm. (j) The SA-β-Gal staining analysis of NHDFs at passage 31 with or without DOX treatment. Bar, 100 μm. (k) The cell culture of *Zmpste24* KO MEF cells and HGPS skin fibroblasts with or without DOX treatment. The red arrows indicate the dying cells. Bar, 200 μm. (l) The PI staining and flow cytometry analysis of cell death of WT MEF cells and NHDFs. (m,n) The western blotting (m) and statistical analysis (n) of relative UPR^mt^ gene expression in live tissues of WT and *Zmpste24* KO mice treated with or without DOX. The red arrow in (m) indicates the bands of prelamin A (lamin A precursor). (o,p) The statistical analysis of relative NAT10 level in WT and *Zmpste24* KO MEF cells (o), HGPS fibroblasts and NHDFs treated with or without DOX (p). n.s., non-significant. * *p* < 0.05.

**Supplementary Table 1 Antibodies used in this study**

| **Antibody** | **Source** | **Dilutions** |
| --- | --- | --- |
|  |  | **WB**, Western blotting;  **IF**, immunofluorescence |
| lamin A/C | Santa Cruz (sc-20681) | WB (1:500); IF (1:100) |
| p21 | Santa Cruz (sc-6246) | WB (1:200) |
| p16INK4a | Proteintech (10883-1-AP) | WB (1:1000 in human fibroblast) |
| p16INK4a | Santa Cruz (sc-1661) | WB (1:200 in MEF) |
| IL6 | Proteintech (21865-1-AP) | WB (1:1000 in human fibroblast) |
| IL6 | CST (12912) | WB (1:1000 in MEF) |
| α-Tubulin | CST (3873S) | WB (1:5000) |
| Ac-α-Tubulin | CST (5335S) | WB (1:1000); IF (1:300) |
| 53BP1 | Abcam (ab14705) | IF (1:300) |
| γH2AX | Millipore (05-636) | IF (1:300) |
| mtCO1 | Abcam (ab14705) | WB (1:1000) |
| LONP1 | Abcam (ab103809) | WB (1:1000) |
| HSP60 | Abcam (ab46798) | WB (1:20000) |
| NAT10 | Proteintech (13365-1-AP) | WB (1:1000) |
| GAPDH | Beyotime (AG019) | WB (1:5000) |

**Supplementary Table 2** **Primers for quantitative PCR**

| **Targets** | **Forward (5’-3’)** | **Reverse (5’-3’)** |
| --- | --- | --- |
| 18s | CGAACGTCTGCCCTATCAACTT | ACCCGTGGTCACCATGGTA |
| mouse GAPDH | AGGTCGGTGTGAACGGATTTG | TGTAGACCATGTAGTTGAGGTCA |
| mouse p16 | CGCAGGTTCTTGGTCACTGT | TGTTCACGAAAGCCAGAGCG |
| mouse p21 | GTGGGTCTGACTCCAGCCC | CCTTCTCGTGAGACGCTTAC |
| mouse IL6 | TAGTCCTTCCTACCCCAATTTCC | TTGGTCCTTAGCCACTCCTTC |
| mouse IL1β | GAAATGCCACCTTTTGACAGTG | TGGATGCTCTCATCAGGACAG |
| human Actin | AAAGACCTGTACGCCAACAC | GTCATACTCCTGCTTGCTGAT |
| human p16 | ATGGAGCCTTCGGCTGACT | GTAACTATTCGGTGCGTTGGG |
| human p21 | TGTCCGTCAGAACCCATGC | AAAGTCGAAGTTCCATCGCTC |
| human IL6 | ACTCACCTCTTCAGAACGAATTG | CCATCTTTGGAAGGTTCAGGTTG |
| human IL1β | ATGATGGCTTATTACAGTGGCAA | GTCGGAGATTCGTAGCTGGA |

**Supplementary Table 3** **Target sequences for human NAT10 shRNA**

| **shRNA** | **Forward (5’-3’)** |
| --- | --- |
| NAT10 sh-1 | CGGCCATCTCTCGCATCTATT |
| NAT10 sh-2 | GCAATTGTACACAGTGACTAT |
